# Efficient Separation of Four Antibacterial Diterpenes from the Roots of *Salvia Prattii* Using Non-Aqueous Hydrophilic Solid-Phase Extraction Followed by Preparative High-Performance Liquid Chromatography

**DOI:** 10.3390/molecules23030623

**Published:** 2018-03-09

**Authors:** Jun Dang, Yulei Cui, Jinjin Pei, Huilan Yue, Zenggen Liu, Weidong Wang, Lijin Jiao, Lijuan Mei, Qilan Wang, Yanduo Tao, Yun Shao

**Affiliations:** 1 Key Laboratory of Tibetan Medicine Research, Northwest Institute of Plateau Biology, Chinese Academy of Sciences, Xining 810008, China; dangjun@nwipb.cas.cn (J.D.); m17701159965@163.com (Y.C.); jinjinpeislg@163.com (J.P.); hlyue@nwipb.cas.cn (H.Y.); lzg2005sk@126.com (Z.L.); wangweidong315@mails.ucas.ac.cn (W.W.); jiaolijin15@mails.ucas.ac.cn (L.J.); meilijuan111@163.com (L.M.); wql@nwipb.cas.cn (Q.W.); 2 Qinghai Provincial Key Laboratory of Tibetan Medicine Research, Xining 810008, China; 3 Shaanxi Key Laboratory of Bio-Resources, Shaanxi University of Technology, Hanzhong 723000, China

**Keywords:** *Salvia prattii*, antibacterial diterpenes, hydrophilic solid-phase extraction, preparative high-performance liquid chromatography

## Abstract

An efficient preparative procedure for the separation of four antibacterial diterpenes from a *Salvia prattii* crude diterpenes-rich sample was developed. Firstly, the XION hydrophilic stationary phase was chosen to separate the antibacterial crude diterpenes-rich sample (18.0 g) into three fractions with a recovery of 46.1%. Then, the antibacterial fractions I (200 mg), II (200 mg), and III (150 g) were separated by the Megress C18 preparative column, and compounds tanshinone IIA (80.0 mg), salvinolone (62.0 mg), cryptotanshinone (70.0 mg), and ferruginol (68.0 mg) were produced with purities greater than 98%. The procedure achieved large-scale preparation of the four diterpenes with high purity, and it could act as a reference for the efficient preparation of active diterpenes from other plant extracts.

## 1. Introduction

*Salvia prattii* (*S. prattii*), acknowledged as an alternative for *Salvia miltiorrhiza*, is extensively utilized in traditional Tibetan medicine. Previous chemical investigations have proved that *Salvia* species possess two main classes of biologically active substances: phenylpropanoids and diterpenes [1,2,3]. Diterpenes, the principal active constituents of other *Salvia* plants, have numerous pharmacological functions, including antibacterial [4], anti-inflammatory [5], and anticancer activities [6,7]. In our preliminary experiment, the crude diterpenes-rich sample of *S. prattii* displayed considerable antibacterial activity against *Staphylococcus aureus* (MIC: 125 μg/mL), *Pseudomonas aeruginosa* (MIC: 125 μg/mL), and *Acinetobacter baumannii* (MIC: 250 μg/mL). To identify the main antibacterial constituents of the sample, it is desirable to obtain the diterpenes in adequate purity and quantity. Thus, the objective of this work is to develop an efficient process for the purification of diterpenes from the diterpenes-rich sample of *S. prattii*.

To date, the separation of diterpenes from *Salvia* plants depends on gel and silica gel open column chromatography [8,9]. However, such methods have numerous drawbacks, such as low yields, being time-consuming, producing a large quantity of solvent waste, and non-suitability for large-scale industrial production. In recent times, high-speed counter-current chromatography, a liquid-liquid chromatographic method, was proposed for the isolation of diterpenes from *Salvia* plants [10,11,12]. Even though this technique offers high-separation efficiency, it requires several hours, rather than minutes needed for preparative high-performance liquid chromatography (prep-HPLC).

Prep-HPLC, is considered as an efficient technique for the separation and purification of phenols, coumarins, flavonoids, and glycosides from intricate mixtures like traditional Tibetan medicines [13,14,15]. It is preferred over other chromatography methods, owing to higher efficiency, greater resolution, and better reproducibility through online monitoring and automatic control [16,17,18]. Consequently, prep-HPLC has been drawing ever-increasing attention from phytochemists and the pharma industry. However, a crude extract cannot be directly subjected to prep-HPLC separation; other separation techniques are usually required for enrichment of the main compounds of the crude extract. Hydrophilic interaction liquid chromatography solid-phase extraction (HILIC-SPE), which employs stationary phases with polar functional groups bonded to silica gel surface, has been extensively used to enrich compounds of interest from natural products due to its applicability, ease of use and regeneration, as well as complementary selectivity to reversed-phase liquid chromatography [19]. A few studies reported the separation of diterpenes from *S. miltiorrhiza* using high-speed counter-current chromatography [20,21,22], but no reports mentioned the separation of diterpenes by a combination of HILIC-SPE and prep-HPLC. Hence, this study aimed to develop a valuable protocol for the purification of four antibacterial diterpenes from a diterpenes-rich *S. prattii* crude sample. The developed protocol succeeded in achieving large-scale preparation of four highly pure diterpenes from the crude sample of *S. prattii*, paving way for the potential development of antibacterial drugs.

## 2. Experimental

### 2.1. Apparatus

The prep-HPLC experiment was performed on a Hanbon DAC-50 prep-HPLC system (Hanbon Science & Technology Co., Ltd., Huai’an, China). The system consisted of a DAC-50 Megress C18 dynamic axial compression column, two prep-HPLC NP7000 pumps, a sample loop of 20.0 mL, a DM-A Dynamic Mixer, a NU3000 UV/Vis detector and an EasyChrom workstation.

The HPLC analysis was carried out on an Agilent 1200 instrument (Agilent Technologies Co., Ltd., Santa Clara, CA, USA) consisting of a G1311A pump, a G1315D UV/Vis detector, a G1316A thermostat, an autosampler and an Agilent workstation. ESI-MS spectra were recorded on an API 2000 mass spectrometer (AB SCIEX, Milwaukee, WI, USA). The NMR spectra were recorded on Bruker Avance 600 MHz (Bruker, Karlsruhe, Germany) spectrometer using tetramethylsilane (TMS) as the internal standard.

### 2.2. Reagents and Stationary Phases

Analytical grade 95% ethanol, *n*-hexane, ethanol, and methanol utilized for the sample extraction, as well as HILIC-SPE and prep-HPLC were ordered from the Tianjin Chemical Factory (Tianjin, China). Chromatographic grade *n*-hexane, ethanol, methanol and acetonitrile employed for the HPLC analysis were bought from Concord Chemical Ltd. (Tianjin, China). Water was purified through a PAT-125 laboratory ultrapure water system from Chengdu ultra Tech (Chengdu, China).

The XION (40–60 μm) and Megress C18 (10 μm) stationary phases were purchased from Acchrom Technologies Co., Ltd. (Beijing, China) and Hanbon Science & Technology Co., Ltd. (Huai’an, China), respectively. The XION (250 mm × 4.6 mm, 40–60 μm) and Megress C18 (250 mm × 4.6 mm, 10 μm) analytical columns were obtained from Acchrom (Beijing, China) and Hanbon Science & Technology Co., Ltd. (Huai’an, China), respectively. Silica gel (250 mm × 4.6 mm, 40–60 μm) and XAqua C3 (250 mm × 4.6 mm, 5 μm) analytical columns were obtained from Acchrom (Beijing, China).

### 2.3. Preparation of the Crude Sample

The roots of *S. prattii* have been obtained from Yushu in Qinghai province, China (September 2016) and authenticated by Prof. Li-Juan Mei of the Northwest Institute of Plateau Biology, Chinese Academy of Sciences. A sample (NWIPB-SPH-2016-11-14) was handed over to Qinghai-Tibetan Plateau Museum of Biology (QPMB). The dried and milled samples (1.2 kg) were extracted thrice for 2 days using 95% ethanol (12.0 L for each extraction) at room temperature. The extracts were combined (36.0 L) and concentrated at 60 °C using a rotary evaporator. The partially dried concentrate (approximately 0.5 L) was suspended in distilled water (2.0 L); the suspension was subsequently loaded onto a preprocessed middle chromatogram isolated gel (MCI) column (10 cm × 100 cm, 2 kg), washed with 40% ethanol (12 L), eluted with of 80% ethanol (12 L) and further dried to yield 18.0 g of crude diterpenes-rich sample for ensuing HILIC-SPE pre-separation.

### 2.4. HILIC-SPE Pre-Separation

The crude diterpenes-rich sample was dissolved in methanol, mixed with polyamide and dried using a rotary evaporator. Afterwards, the solid mixture was loaded onto a XION solid-phase extraction medium-pressure column (300 mm × 50 mm, containing 297.7 g solid-phase XION) and eluted with four column volumes of *n*-hexane/ethanol (20:0, 19:1, 18:2, 16:4 and 14:6 *v*/*v*), successively. The eluent from the HILIC-SPE column was collected in 100 mL fractions and analyzed by HPLC using a XION (250 mm × 4.6 mm, 40–60 μm) analytical column. The eluents with the same composition were collected and combined according to the HPLC analysis. Finally, the fractions eluted with *n*-hexane/ethanol 16:4 and 14:6 *v*/*v* gave fraction I (2.8 g), fraction II (3.4 g), and fraction III (2.1 g), respectively. The three fractions were stored in a refrigerator for subsequent preparative separation.

### 2.5. Antibacterial Activity

*Staphylococcus aureus* (ATCC 25923), *Pseudomonas aeruginosa* (ATCC 27853), and *Acinetobacter baumannii* (obtained from the People’s Liberation Army (PLA) General Hospital) were used as the instruction strains for the antibacterial activity assay. Mueller-Hinton broth was used to culture bacteria and an increase in optical density at 600 nm was used to monitor growth. The two-fold serial dilutions of the active extracts and diterpenes (dissolved in dimethyl sulfoxide, DMSO) were added into the sensitive strains, respectively. The minimum inhibitory concentration (MIC), defined as the lowest concentration of the active extracts and diterpenes needed to inhibit the growth of the sensitive strains, was observed following incubation at 30 °C for 18 h according to the Clinical and Laboratory Standards Institute (CLSI, Wayne, PA, USA, 2008). The mid-exponential broth of sensitive strains treated without the extracts, diterpenes and DMSO were considered as the negative control. The growth of only DMSO-treated sensitive strains was monitored to eliminate the effect of DMSO. The mid-exponential broth of sensitive strains treated with the antibiotic cefotaxime and vancomycin (1.0 mg/mL) were used as positive controls.

### 2.6. Purification of the Main Diterpenes by Prep-HPLC

The purification of diterpenes was performed on a Hanbon DAC-50 prep-HPLC system. Fractions I, II and III were dissolved in methanol and injected onto a DAC-50 dynamic axial compression column containing the Megress C18 stationary phase (flow rate: 60 mL/min; injection volume: 5.0 mL). The mobile phases consisted of 0.2% *v*/*v* formic acid in water and 0.2% *v*/*v* formic acid in methanol at different ratios (15:85 for fraction I, 20:80 for fractions II and III). The effluent was analyzed using a UV/Vis detector at 254 nm and was manually obtained based on the chromatograms. The collected fractions were subsequently evaporated to dryness in reduced pressure at 60 °C.

### 2.7. HPLC Analysis and Identification of the Separated Diterpenes

HPLC analysis of the separated diterpenes was carried out at 25 °C, on a XAqua C3-column (flow rate: 1.0 mL/min) and the chromatogram was recorded at 254 nm. Water and methanol were the mobile phases A and B, respectively. The gradient elution steps were as follows: 0–30 min, 75–85% B.

The chemical structures of the separated diterpenes were established by UV, Mass, ^1^H-NMR and ^13^C-NMR spectrometry. The UV spectra were recorded using the DAD detector of the Agilent 1200 system. ESI-MS spectra were recorded on an API 2000 mass spectrometer in positive ion mode, whereas NMR spectra were recorded on a Bruker Avance III 600 MHz spectrometer.

## 3. Results and Discussion

### 3.1. HILIC-SPE Column Chromatography Fractionation and Antibacterial Activity Screening

**Figure 1:**
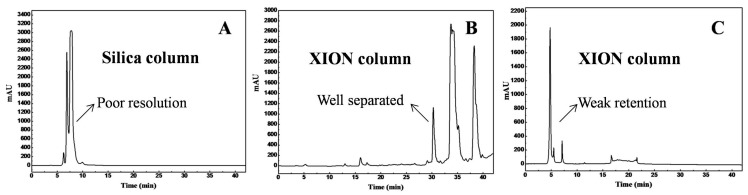
Representative analytical chromatograms of *S. prattii* crude extract on a silica analytical column (**A**) and XION analytical column (**B**,**C**). The conditions of (**A**,**B**) were the same: mobile phase A: *n*-hexane, and mobile phase B: ethanol; gradient: 0–8.4 min, 0% B; 8.4–16.8 min, 5%~5% B; 16.8–25.2 min, 10%~10% B; 25.2–33.6 min, 20%~20% B; 33.6–42 min, 30%~30% B. Conditions of (**C**): mobile phase A: 0.2% *v*/*v* formic acid in acetonitrile, and mobile phase B: 0.2% *v*/*v* formic acid in water; gradient: 0–10 min, 0%~0% B; 10~56 min, 0%~95% B. The other conditions were the same: monitoring wavelength: 254 nm; flow rate: 1 mL/min; injection volume: 10 μL; column temperature: 25 °C.

To simplify the development of the reversed-phase prep-HPLC method and to improve the life-span of the reversed-phase stationary phase, the crude diterpenes-rich extract with complex composition usually requires pretreatment. To select an appropriate pretreatment, two chromatographic stationary phases i.e., the bare silica gel and the XION stationary phases, in three separation modes were tested for the separation of the crude extract; the representative separation chromatograms are shown in Figure 1. As observed in Figure 1A,B, the main diterpenes had inferior resolution on the bare silica gel stationary phase compared to that on the XION stationary phase under the same elution conditions (*n*-hexane/ethanol solvent system). The main diterpenes showed weak retention on the XION stationary phase with the mobile phases of 0.2% *v*/*v* formic acid in acetonitrile and in water (Figure 1C). According to the manufacturer, XION is a cysteine-bond silica gel stationary phase, and cysteine is a polar group, which gives the XION stationary phase the retention behavior of normal-phase chromatography and hydrophilic interaction chromatography [23,24]. Thus, the XION stationary phase should be employed for sample pretreatment under the normal-phase mode due to the favorable separation profile (Figure 1B).

An analytical column (250 mm × 4.6 mm, 40–60 μm) for hydrophilic interaction chromatography is usually packed with 2.1 g of stationary phase (ρ was approximately 0.5 g/mm^3^ under the conditions of high pressure), and one column volume is 2.1 mL (one column volume: weight of the stationary phase = 1 mL:1 g). The calculations used the following equation:

(1) $$\frac{\rho_{A}\pi R_{A}^{2}H_{A}}{\rho_{A}\pi R_{P}^{2}H_{P}}=\frac{m_{A}}{m_{P}}$$

where ρ_A_ and ρ_P_ are stationary phase packing densities of the analytical column and HILIC-SPE column (under the conditions of high pressure, ρ_A_ = ρ_P_), respectively; R_A_ and R_P_ are the diameters of the analytical column (4.6 mm) and HILIC-SPE column (50 mm), respectively; H_A_ and H_P_ are the column lengths of the analytical column (250 mm) and HILIC-SPE column (actual packing length was 300 mm), respectively; similarly, m_A_ and m_P_ are stationary phase weights of the analytical column and the HILIC-SPE column, respectively. For the same stationary phase, the packing density in the analytical column and HILIC-SPE column was uniform under the conditions of high pressure. Thus, the above equation could be simplified as:

(2) $$\frac{R_{A}^{2}H_{A}}{R_{P}^{2}H_{P}}=\frac{m_{A}}{m_{P}}$$

**Figure 2:**
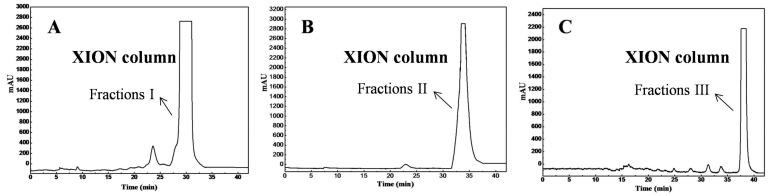
The analytical chromatograms of target fractions I (**A**), II (**B**) and III (**C**) on a XION analytical column. Conditions of (**A**), (**B**), and (**C**): mobile phase A: *n*-hexane, and mobile phase B: ethanol; gradient: 0–8.4 min, 0%~0% B; 8.4–16.8 min, 5%~5% B; 16.8–25.2 min, 10%~10% B; 25.2–33.6 min, 20%~20% B; 33.6–42 min, 30%~30% B. Other conditions: monitoring wavelength: 254 nm; flow rate: 1 mL/min; injection volume: 10 μL; column temperature: 25 °C.

The calculations showed that the HILIC-SPE column (300 mm × 50 mm, 40–60 μm) should be packed with 297.7 g of stationary phase, and one column volume is 297.7 mL. Therefore, the sample loaded onto the XION solid-phase extraction column was eluted with 1190.8 mL (four column volumes) of *n*-hexane/ethanol (20:0, 19:1, 18:2, 16:4, and 14:6 *v*/*v*, successively); the same gradient elution was used for the XION analytical column and HILIC-SPE column. The HPLC analysis results revealed that the diterpene fractions were mainly present in the eluates of the 16:4 and 14:6 *v*/*v n*-hexane/ethanol. Figure 2 shows the target fractions I (Figure 2A), II (Figure 2B), and III (Figure 2C). By comparing the Figure 1 and Figure 2, it could be seen that the diterpenes were divided into three groups (fractions I, II and III) with the *n*-hexane/ethanol mobile phase on the XION stationary phase. Following the HILIC-SPE column chromatography, 2.8 g of fraction I, 3.4 g of fraction II, and 2.1 g of fraction III were obtained from 18.0 g of the antibacterial crude diterpenes-rich sample.

The antibacterial activities (MIC in μg/mL) of the tested fractions I–III are shown in Table 1. Fractions I–III displayed higher antimicrobial activity compared to the crude diterpenes-rich sample against *Staphylococcus aureus*, *Pseudomonas aeruginosa,* and *Acinetobacter baumannii*. Therefore, it is of interest to identify the antibacterial compounds from the bioactive fractions I, II, and III through further separation.

### 3.2. Purification of Diterpenes by Reversed-Phase Preparative High-Performance Liquid Chromatography

To attain a good separation profile, the stationary phases used in the analytical HPLC and preparative liquid chromatography were the same. The elution conditions of fractions I, II, and III were standardized on the Megress C18 analytical column, whereas the linear magnifying methodology has been utilized to transform the analytical flow rate to the preparative level using the following equation:

(3) $$\frac{R_{A}^{2}}{R_{P}^{2}}=\frac{F_{A}}{F_{P}}$$

where R_A_ and R_P_ are the diameters of the analytical column (4.6 mm) and preparative column (50 mm), respectively. Similarly, F_A_ and F_P_ are the flow rates of the analytical column and preparative column, respectively. For convenience, the isocratic elution conditions of same sample solutions were optimized on the Megress C18 analytical column (250 mm × 4.6 mm, 10 μm) at a flow rate of 0.5 mL/min (F_A_). The comparable flow rate in the Megress C18 preparative column (250 mm × 50 mm, 10 μm) has been determined to be 59.1 mL/min (F_P_), hence, 60.0 mL/min was used for convenience. For the loading amount, a similar linear magnifying technology was employed with the equation:

(4) $$\frac{R_{A}^{2}}{R_{P}^{2}}=\frac{M_{A}}{M_{P}}$$

For the same concentration (40.0 mg/mL for fractions I and II; 30.0 mg/mL for fraction III) of the sample solutions on the analytical and preparative columns, the equation could be simplified as:

(5) $$\frac{R_{A}^{2}}{R_{P}^{2}}=\frac{V_{A}}{V_{P}}$$

where M_A_ and M_P_ are the loading amounts of the analytical and preparative columns, respectively. Similarly, V_A_ and V_P_ are the injection volumes of the analytical and preparative columns, respectively. The injection volumes (loading amount) of the analytical column and the preparative columns were 10 μL (V_A_) and1181.5 μL (1.181 mL), respectively. However, this volume is too low to meet the requirements for the highly proficient preparation, and the preparative injection volume was larger but smaller than that of the sample loop (5.0 mL for fractions I, II, and III in actual operation).

**Figure 3:**
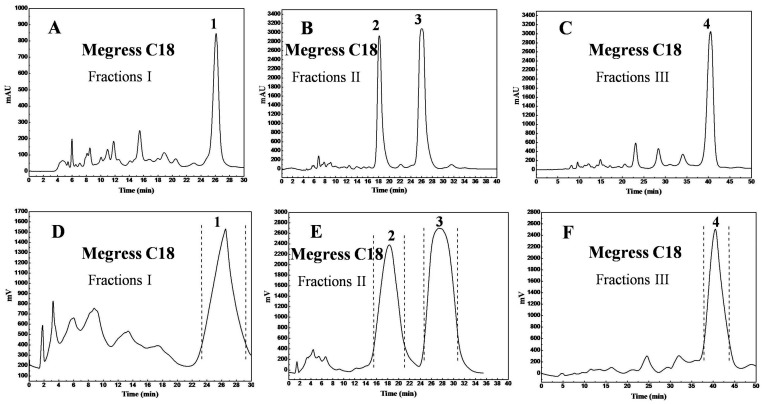
Analytical chromatograms of target fractions I (**A**), II (**B**), and III (**C**) on a Megress C18 analytical column and preparative chromatograms of target fractions I (**D**), II (**E**), and III (**F**) on a Megress C18 preparative column. The mobile phases for fractions I (**A**,**D**), II (**B**,**E**), and III (**C**,**F**) were the same: A: 0.2% *v*/*v* formic acid in water, and B: 0.2% *v*/*v* formic acid in methanol. Isocratic: 0–30 min, 85%~85% B for fraction I (**A**,**D**). Isocratic: 0–40 min, 80%~80% B for fraction II (**B**,**E**). Isocratic: 0–50 min, 80%~80% B for fraction III (**C**,**F**). The other conditions were the same: monitoring wavelength: 254 nm; flow rate: 1 mL/min; injection volume: 10 μL; column temperature: room temperature.

Under the optimized conditions, the separation chromatograms of fractions I–III on the analytical level and preparative level were shown in Figure 3. The optimized analytical (Figure 3A–C) and preparative (Figure 3D–F) chromatographic profiles were similar, except for a slight increase in the retention times of peaks 1–4 on the prep-chromatograms. For peaks 2 and 3 (Figure 3B,E), the resolution on the analytical and preparative chromatograms were R_SA_ = 2(R_t2_ − R_t1_)/(W_t2_ + W_t1_) = 2.55 and R_SP_ = 1.2, respectively, due to sample-overloading and the preparative column diffusion effect. The target compound chromatographic peaks were collected based on the UV absorption intensities considerably enhance the purity. Overall, 200.0 mg each of fractions I and II as well as 150.0 mg of fraction III were separated to obtain 80.0 mg of the peak 1 fraction, 62.0 mg of the peak 2 fraction, 70.0 mg of the peak 3 fraction, and 68.0 mg of the peak 4 fraction with recoveries of 40.0%, 66.0%, and 45.3%, respectively.

**Figure 4:**
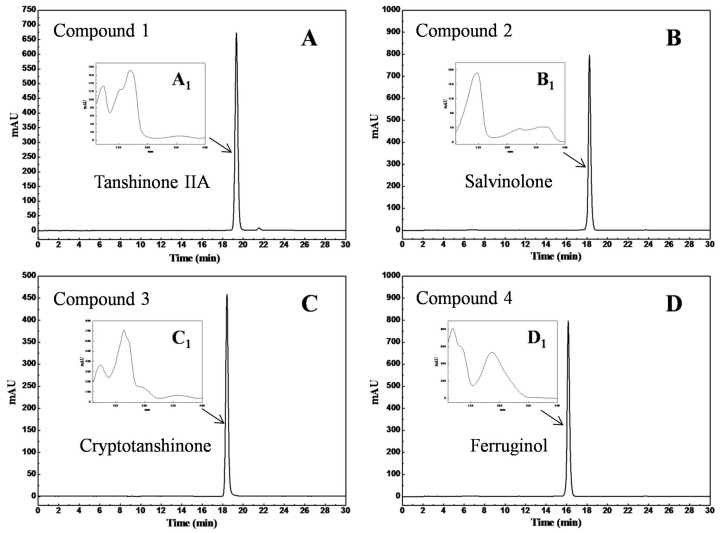
Purity analysis chromatograms (**A**–**D**) of the isolated compounds on an XAqua C3 analytical column and their UV spectra (**A_1_**–**D_1_**). Conditions: mobile phase A: water, and mobile phase B: methanol; gradient: 0–30 min, 75%~85% B. Other conditions: monitoring wavelength: 254 nm; flow rate: 1.0 mL/min; injection volume: 10 μL; column temperature: 25 °C.

The purities of the four isolated diterpenes were determined by HPLC on XAqua C3 analytical column (250 mm × 4.6 mm, 5 μm). The results are given in Figure 4: Figure 4A–D show that the compounds possess purity greater than 98% (98.6% for compound **1**, 99.9% for compound **2**, 99.1% for compound **3**, and 99.7% for compound **4**). The UV spectra of the four diterpenes are included in the insets of Figure 4 (Figure 4A_1_–D_1_); they were in accordance with those in previous publications [23,24,25,26]. In addition, the antibacterial activity of the four isolated diterpenes was also tested (Table 1): Compound **4** showed the strongest activity (MIC: 10–15 μg/mL) against *Staphylococcus aureus*, *Pseudomonas aeruginosa,* and *Acinetobacter baumannii*; whereas compounds **1**–**3** also displayed potent activity against the same species (MIC: 20–50 μg/mL).

**Figure 5:**
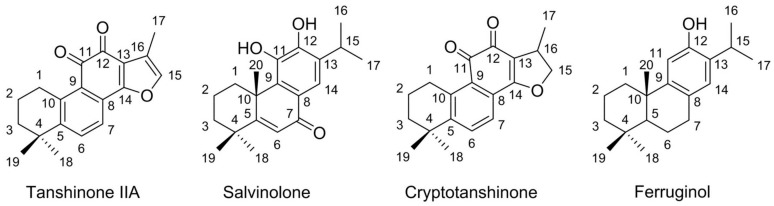
The chemical structures of tanshinone IIA (compound **1**), salvinolone (**2**), cryptotanshinone (**3**), and ferruginol (**4**).

The chemical structures of the isolated diterpenes have been determined by ESI-MS, ^1^H-NMR, and ^13^C-NMR spectroscopy. By comparing the spectra of the compounds with literature data, we concluded that compounds **1**–**4** represent tanshinone IIA, salvinolone, cryptotanshinone, and ferruginol. The chemical structures of tanshinone IIA (compound **1**), salvinolone (**2**), cryptotanshinone (**3**), and ferruginol (**4**) are shown in Figure 5 and the detailed data of the compounds are given below.

Compound **1**: Red powder, ESI-MS *m*/*z*: 295.3 [M + H]^+^. ^1^H-NMR (600 MHz, CDCl_3_): δ 7.63 (1H, d, *J* = 8.1 Hz, H-6), 7.55 (1H, d, *J* = 8.1 Hz, H-7), 7.22 (1H, s, H-16), 3.18 (2H, t, *J* = 6.4 Hz, H-1), 2.26 (3H, s, H-17), 1.79 (2H, m, H-2), 1.66 (2H, m, H-3), 1.31 (6H, s, H-18, 19); ^13^C-NMR (151 MHz, CDCl_3_): 183.8 (C-11), 175.9 (C-12), 161.9 (C-14), 150.3 (C-10), 144.6 (C-5), 141.4 (C-15), 133.6 (C-6), 127.6 (C-8), 126.6 (C-9), 121.3 (C-16), 120.4 (C-7), 120.0 (C-13), 38.0 (C-3), 34.8 (C-4), 32.0 (C-18), 32.0 (C-19), 30.0 (C-1), 19.3 (C-2), 9.0 (C-17). The ESI-MS, ^1^H-NMR and ^13^C-NMR data for compound **1** agree with the literature data reported for tanshinone IIA [25].

Compound **2**: Red powder, ESI-MS *m*/*z*: 315.3 [M + H]^+^. ^1^H-NMR (600 MHz, CDCl_3_): δ 7.72 (1H, s, H-14), 6.45 (1H, s, H-6), 3.28 (2H, m, H-15), 1.67 (3H, s, H-20), 1.36 (3H, s, H-18), 1.32 (3H, d, *J* = 6.5 Hz, H-17), 1.30 (3H, d, *J* = 6.5 Hz, H-16), 1.26 (3H, s, H-19); ^13^C-NMR (151 MHz, CDCl_3_): 185.8 (C-7), 175.2 (C-5), 144.6 (C-12), 141.5 (C-11), 137.5 (C-9), 132.6 (C-13), 124.4 (C-6), 123.7 (C-8), 115.8 (C-14), 42.3 (C-10), 40.5 (C-3), 38.3 (C-4), 34.5 (C-1), 33.3 (C-20), 29.6 (C-19), 27.6 (C-18), 25.1 (C-15), 22.8 (C-16), 22.6 (C-17), 18.9 (C-2). The ESI-MS, ^1^H-NMR and ^13^C-NMR data for compound **2** agree with the literature data reported for salvinolone [26].

Compound **3**: Orange powder, ESI-MS *m*/*z*: 297.4 [M + H]^+^. ^1^H-NMR (600 MHz, CDCl_3_): δ 7.63 (1H, d, *J* = 8.1 Hz, H-6), 7.49 (1H, d, *J* = 8.1 Hz, H-7), 4.88 (1H, t, *J* = 9.4 Hz, H-15), 4.36 (1H, dd, *J* = 9.2 and 6.0 Hz, H-16), 3.21 (2H, t, *J* = 6.4 Hz, H-1), 1.78 (2H, m, H-2), 1.65 (2H, m, H-3), 1.35 (3H, d, *J* = 6.8 Hz, H-17), 1.30 (6H, s, H-18, 19); ^13^C-NMR (151 MHz, CDCl_3_): 184.4 (C-11), 175.8 (C-12), 171.0 (C-14), 152.5 (C-10), 143.8 (C-5), 132.7 (C-6), 128.5 (C-8), 126.3 (C-9), 122.6 (C-7), 118.4 (C-13), 81.6 (C-15), 37.9 (C-3), 35.0 (C-4), 34.7 (C-16), 32.0 (C-18), 32.0 (C-19), 29.8 (C-1), 19.2 (C-2), 19.0 (C-17). The ESI-MS, ^1^H-NMR and ^13^C-NMR data for compound **3** agree with the literature data reported for cryptotanshinone [27].

Compound **4**: Yellow powder, ESI-MS *m*/*z*: 287.4 [M + H]^+^. ^1^H-NMR (600 MHz, CDCl_3_): δ 6.84 (1H, s, H-14), 6.63 (1H, s, H-10), 3.13 (1H, m, H-15), 2.87 (1H, dd, *J* = 16.6 and 6.6 Hz, H-7a), 2.77 (1H, m, H-7b), 1.25 (3H, d, *J* = 7.4 Hz, H-17), 1.23 (3H, d, *J* = 7.2 Hz, H-16), 1.18 (1H, s, H-20), 0.95 (3H, s, H-19), 0.93 (3H, s, H-18); ^13^C-NMR (151 MHz, CDCl_3_): 150.8 (C-12), 148.8 (C-9), 131.5 (C-13), 127.4 (C-8), 126.7 (C-14), 111.1 (C-11), 50.5 (C-5), 41.8 (C-3), 39.0 (C-1), 37.6 (C-10), 33.6 (C-4), 33.5 (C-18), 29.9 (C-7), 26.9 (C-15), 24.9 (C-20), 22.9 (C-17), 22.7 (C-16), 21.8 (C-19), 19.5 (C-2), 19.4 (C-6). The ESI-MS, ^1^H-NMR and ^13^C-NMR data for compound **4** agree with the literature data reported for ferruginol [28].

## 4. Concluding Remarks

An efficient preparative procedure involving non-aqueous HILIC-SPE followed by prep-HPLC was developed for the preparation of antibacterial tanshinone IIA, salvinolone, cryptotanshinone, and ferruginol from a crude diterpenes-rich sample of the roots of *S. prattii*. Initially, HILIC-SPE was used to separate 18.0 g of crude diterpenes-rich sample into three fractions (2.8 g of fraction I, 3.4 g of fraction II, and 2.1 g of fraction III). Then, a DAC-50 prep-HPLC column containing Megress C18 stationary phase was used to isolate the diterpenes from the three fractions. The separation of each fraction on the Megress C18 was performed by optimizing the separation conditions on the Megress C18 analytical column and transforming the conditions to a Megress C18 preparative column. The coeluted diterpenes from the HILIC-SPE stationary phase were separated at large-scale sample amounts on the Megress C18 reversed stationary phase, brought about by the diverse sample separation mechanisms of both the stationary phases. Owing to the different selectivities and the optimized collection mode, 80.0 mg of tanshinone IIA, 62.0 mg of salvinolone, 70.0 mg of cryptotanshinone, and 68.0 mg of ferruginol at greater than 98% purity were prepared from 200.0 mg each of fractions I and II as well as 150.0 mg of fraction III with a single preparation. The purified diterpenes were identified as tanshinone IIA, salvinolone, cryptotanshinone, and ferruginol by UV, Mass, and NMR spectroscopy. Therefore, the present work offers an excellent protocol for the preparation of active diterpenes from plant sources.

## Figures and Tables

**Table 1:** Antimicrobial activity of the crude diterpene-rich sample, fractions and, compounds (MIC in μg/mL).

Bacteria					Cf
Crude diterpene-rich sample					
*Staphylococcus aureus*	125				0.5
*Pseudomonas aeruginosa*	125				7.5
*Acinetobacter baumannii*	250				12.5
Fractions I, II and III					
	I	II	III		
*Staphylococcus aureus*	100	125	50		0.5
*Pseudomonas aeruginosa*	100	150	50		7.5
*Acinetobacter baumannii*	150	150	50		12.5
Compounds					
	**1**	**2**	**3**	**4**	
*Staphylococcus aureus*	25	30	25	10	0.5
*Pseudomonas aeruginosa*	20	50	30	15	7.5
*Acinetobacter baumannii*	25	50	25	15	12.5

(**1**) tanshinone IIA; (**2**) salvinolone; (**3**) cryptotanshinone; (**4**) ferruginol. Cf: Vancomycin for *Staphylococcus aureus*; cefotaxime for *Pseudomonas aeruginosa* and *Acinetobacter baumannii*.
